# Understanding and Measuring Frailty: Insights From the Canadian NuAge and CLSA Cohorts

**DOI:** 10.1093/geroni/igab046.1478

**Published:** 2021-12-17

**Authors:** Pierrette Gaudreau, Alan Cohen

## Abstract

Frailty is one of the most central concepts in geriatrics; nonetheless, multiple definitions and operationalizations abound, and the underlying biology remains a topic of much discussion. Here, we bring together four talks that join questions of understanding with questions of measurement, in order to explore how answering each is necessary to make progress on the other. We cannot measure frailty if we have not understood and defined it, but we cannot understand if we cannot measure it and study it. Turcot et al. present work on operationalizing frailty in the NuAge cohort. Mayo et al. establish a scale to test the extent to which frailty can be operationalized as a ladder rather than a condition, again using the NuAge cohort. Mendo et al. use mediation analyses to understand how grip strength and other aspects of frailty may play a role in the relationship between diabetes and atherosclerosis. Ghachem et al. test the relationship between physiological dysregulation of different systems and different criteria of the Fried model, in order to assess the evidence for frailty as an emergent physiological state. Together, these talks will push the boundaries of how we think about frailty at levels ranging from biological to clinical to operational.

